# Factors Associated With Racial and Ethnic Disparities in Locally Advanced Rectal Cancer Outcomes

**DOI:** 10.1001/jamanetworkopen.2024.0044

**Published:** 2024-02-29

**Authors:** Rebecca M. Shulman, Mengying Deng, Elizabeth A. Handorf, Joshua E. Meyer, Shannon M. Lynch, Sanjeevani Arora

**Affiliations:** 1 Department of Radiation Oncology, Fox Chase Cancer Center, Philadelphia, Pennsylvania; 2 Biostatistics and Bioinformatics Program, Fox Chase Cancer Center, Philadelphia, Pennsylvania; 3 Cancer Prevention and Control Program, Fox Chase Cancer Center, Philadelphia, Pennsylvania

## Abstract

**Importance:**

Hispanic and non-Hispanic Black patients receiving neoadjuvant therapy and surgery for locally advanced rectal cancer (LARC) achieve less favorable clinical outcomes than non-Hispanic White patients, but the source of this disparity is incompletely understood.

**Objective:**

To assess whether racial and ethnic disparities in treatment outcomes among patients with LARC could be accounted for by social determinants of health and demographic, clinical, and pathologic factors known to be associated with treatment response.

**Design, Setting, and Participants:**

The National Cancer Database was interrogated to identify patients with T3 to T4 or N1 to N2 LARC treated with neoadjuvant therapy and surgery. Patients were diagnosed between January 1, 2004, and December 31, 2017. Data were culled from the National Cancer Database from July 1, 2022, through December 31, 2023.

**Exposure:**

Neoadjuvant therapy for rectal cancer followed by surgical resection.

**Main Outcomes and Measures:**

The primary outcome was the rate of pathologic complete response (pCR) following neoadjuvant therapy. Secondary outcomes were rate of tumor downstaging and achievement of pN0 status.

**Results:**

A total of 34 500 patient records were reviewed; 21 679 of the patients (62.8%) were men and 12 821 (37.2%) were women. The mean (SD) age at diagnosis was 59.7 (12.0) years. In terms of race and ethnicity, 2217 patients (6.4%) were Hispanic, 2843 (8.2%) were non-Hispanic Black, and 29 440 (85.3%) were non-Hispanic White. Hispanic patients achieved tumor downstaging (48.9% vs 51.8%; *P* = .01) and pN0 status (66.8% vs 68.8%; *P* = .02) less often than non-Hispanic White patients. Non-Hispanic Black race, but not Hispanic ethnicity, was associated with less tumor downstaging (odds ratio [OR], 0.86 [95% CI, 0.78-0.94]), less frequent pN0 status (OR, 0.91 [95% CI, 0.83-0.99]), and less frequent pCR (OR, 0.81 [95% CI, 0.72-0.92]). Other factors associated with reduced rate of pCR included rural location (OR, 0.80 [95% CI, 0.69-0.93]), lack of or inadequate insurance (OR for Medicaid, 0.86 [95% CI, 0.76-0.98]; OR for no insurance, 0.65 [95% CI, 0.54-0.78]), and treatment in a low-volume center (OR for first quartile, 0.73 [95% CI, 0.62-0.87]; OR for second quartile, 0.79 [95% CI, 0.70-0.90]; OR for third quartile, 0.86 [95% CI, 0.78-0.94]). Clinical and pathologic variables associated with a decreased pCR included higher tumor grade (OR, 0.58 [95% CI, 0.49-0.70]), advanced tumor stage (OR for T3, 0.56 [95% CI, 0.42-0.76]; OR for T4, 0.30 [95% CI, 0.22-0.42]), and lymph node–positive disease (OR for N1, 0.83 [95% CI, 0.77-0.89]; OR for N2, 0.73 [95% CI, 0.65-0.82]).

**Conclusions and Relevance:**

The findings of this cohort study suggest that disparate treatment outcomes for Hispanic and non-Hispanic Black patients are likely multifactorial in origin. Future investigation into additional social determinants of health and biological variables is warranted.

## Key Points

**Question:**

Do social determinants of health and demographic, clinical, and pathologic factors account for the racial and ethnic disparities observed among patients treated with neoadjuvant therapy and surgery for locally advanced rectal cancer (LARC)?

**Findings:**

In this cohort study of 34 500 patients, treatment outcomes for LARC were less favorable for Hispanic and non-Hispanic Black patients than for non-Hispanic White patients. After accounting for all study variables, non-Hispanic Black race remained independently associated with a less favorable treatment response.

**Meaning:**

These findings suggest that racial and ethnic disparities in LARC treatment outcomes may be multifactorial, with an independent association with non-Hispanic Black race, suggesting unidentified biological variables or social determinants of health that warrant exploration.

## Teaser

This cohort study investigates social determinants of health and prognostically relevant demographic, clinical, and pathologic variables to assess racial and ethnic disparities in the treatment of patients with locally advanced rectal cancer.

## Introduction

In approximately 60% of cases, rectal cancer is locally advanced (stage II or III) at diagnosis and is treated under current guidelines with chemoradiotherapy and surgery.^1,2^ Although the frequency of locally advanced rectal cancer (LARC) among elderly patients in the US has declined, patients younger than 50 years have experienced both an increased frequency of LARC and a disproportionate incidence of higher-stage cancers.^3,4,5,6,7^ Demographic data reveal that young Black and Hispanic patients account for much of this trend.^8^ Furthermore, the racial and ethnic disparities seen in LARC frequency have been accompanied by a similar but poorly understood disparity in LARC survival.^9,10,11,12,13,14^ Black patients with LARC, in particular, have been shown to be approximately 45% more likely to die of their disease than White patients.^15^

Several potential contributors to racial disparities in treatment outcomes are well-established. For example, Black patients are less likely to receive adjuvant chemotherapy or radiotherapy for rectal cancer than White patients, even after controlling for age, clinical stage at diagnosis, and comorbid disease.^11,16^ Large-scale studies of LARC^17,18^ have also established the role of pathologic factors—tumor stage, lymph node status, and circumferential resection margin—in determining overall LARC survival, suggesting a potential role for these variables in any investigation of disparate outcomes. More recently, retrospective studies of LARC^19,20^ have demonstrated the contribution to racial disparities of social determinants of health (SDOH), including educational level, income, marital status, geographic location, and access to treatment. Indeed, racial and ethnic minority individuals have been significantly underrepresented in clinical trials, and 43% of US cancer studies omit altogether the classification of outcome data by racial groups.^21,22^

Five main domains of SDOH are associated with racial or ethnic disparities and poor cancer outcomes: economic stability, educational attainment, health and health care access (eg, insurance status, health literacy), neighborhood and built environment (eg, neighborhood socioeconomic status, urban vs rural environment), and social and community context (eg, social support, perceived discrimination).^23,24,25,26,27,28^ Historically, if studies examining LARC have incorporated SDOH, it has been to a limited extent.^19,20,24,29,30,31,32,33,34,35^ As an illustration, a recent study of patients with colorectal cancer undergoing surgery^36^ demonstrated decreased 1- and 5-year survival for Asian, Hispanic, non-Hispanic Black, and Pacific Islander patient populations compared with White patients. This disparity persisted after adjustment for multiple demographic variables, including insurance status and marital status, with Hispanic patients exhibiting the worst survival decrement.^36^ However, multiple additional factors are evidently at work.

Herein, we have undertaken an analysis using a focused inventory of SDOH in addition to prognostically relevant demographic, clinical, and pathologic data within a contemporary National Cancer Database (NCDB) cohort. Accordingly, we have focused on the pathologic complete response (pCR), which is considered a favorable outcome and an important prognostic factor for patients with rectal cancer,^37,38^ as a key measure of disparate outcomes for non-Hispanic Black and Hispanic patients with LARC who undergo neoadjuvant therapy and surgery.

## Methods

### Data Source and Study Sample

**Figure 1.**
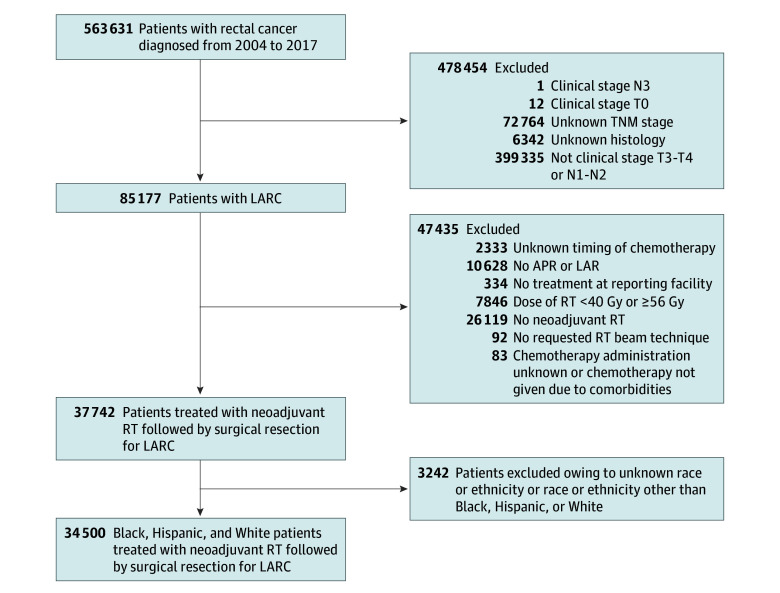
Study Flow Diagram APR indicates abdominoperineal resection; LAR, low anterior resection; LARC, locally advanced rectal cancer; and RT, radiotherapy.

Data for patients with LARC were culled from the NCDB. All work was conducted from July 1, 2022, through December 31, 2023. Patients were eligible for the study if they were 18 years or older, diagnosed with T3 to T4 or N1 to N2 rectal cancer between January 1, 2004, and December 31, 2017, and treated with neoadjuvant therapy followed by surgical resection (Figure 1). Exclusion criteria (Figure 1) included missing race and ethnicity data, clinical or histologic data, unknown timing of chemotherapy, and total radiation dose that is less than or greater than standard conventional dosing (<40 or ≥56 Gy).^39,40,41^ This study followed the reporting requirements of the Strengthening the Reporting of Observational Studies in Epidemiology (STROBE) Statement. In accordance with Fox Chase Cancer Center Institutional Review Board guidelines, interrogation of the deidentified NCDB was not considered human participant research and therefore did not require informed consent.

### Outcome Measures

Pathologic complete response served as the primary outcome, defined to be a T0 N0 surgical specimen obtained following neoadjuvant therapy. Patients with a pCR after neoadjuvant therapy have improved survival, reduced local recurrence, and improved odds for sphincter preservation at surgery.^37,42,43,44,45,46^ Secondary outcomes were tumor downstaging and pN0 status. Tumor downstaging, defined to be a specimen-derived pathologic stage lower than the clinical tumor stage assessed before neoadjuvant therapy, is an important indicator of treatment response and is associated with improved outcomes, including increased eligibility for less radical surgery or nonoperative management.^47,48,49^ Pathologic N0 status was defined as a lymph node–negative surgical specimen and is associated with a lower risk of recurrence and improved survival.^48,49^

### Demographic, Clinical, and Pathologic Covariates and SDOH

Patient information extracted from the NCDB Participant User File included age at diagnosis (<40, 40-49, 50-59, 60-69, or ≥70 years), sex (male or female), type of surgery (low anterior resection [LAR] or abdominoperineal resection [APR]), dose of radiation (<45.0, 45.0-50.4, or >50.4 Gy), neoadjuvant chemotherapy (yes or no), surgical margins (positive or negative), tumor grade (1-4 or unknown), tumor stage (T0-T4), nodal stage (N0-N2), and race and ethnicity. To distinguish between staging at diagnosis and subsequent downstaging, we used the standard approach. We used the initial tumor staging and grade information provided in the NCDB data,^50,51^ which represents the stage of the cancer at the time of diagnosis. Downstaging was assessed by comparing the initial staging (at diagnosis) with the final pathologic staging following neoadjuvant therapy and surgical resection. Downstaging was determined by evaluating changes in tumor size, lymph node involvement, and other relevant factors between the initial diagnosis and posttreatment surgical pathology reports. The outcome assessments were based on data available at the time of surgical resection, after total neoadjuvant therapy. The timing of this assessment varies among patients but generally occurs within a specific time frame after the completion of neoadjuvant therapy, which can range from 4 to 12 weeks.^52^

Race and ethnicity were categorized using self-reported data in the NCDB Participant User File. Patients who self-reported Hispanic ethnicity were classified as Hispanic regardless of self-reported race. Patients missing both race and ethnicity data were not included in the study. This coding process resulted in 3 race and ethnic groups for comparison: Hispanic, non-Hispanic Black, and non-Hispanic White. Measures of SDOH include medical insurance (Medicare, Medicaid, private, or uninsured), facility location (large metropolitan, small metropolitan, suburban, rural, or unknown), hospital volume quartile (1-7, 8-16, 17-35, or ≥36 total LARC cases), comorbidities (yes or no), and area-level measures (linked from the 2012 American Community survey based on patient’s residential zip codes at diagnosis), including median income (grouped high or low based on combining quartiles) and educational level (grouped high or low based on quartiles).

### Statistical Analysis

Patients were categorized as Hispanic, non-Hispanic Black, or non-Hispanic White based on self-reported race and ethnicity. The demographic and clinical data for the 3 study groups were compared using analysis of variance and χ^2^ tests, with additional pairwise tests. The response to neoadjuvant therapy was determined by the rates achieved for pCR, tumor downstaging, and pN0 status. Multivariate logistic regression models were used to investigate the association between race and ethnicity groups adjusting for SDOH and demographic and clinical factors. To account for the correlation within facilities, generalized estimating equations with robust SEs were used. As a sensitivity analysis, we conducted additional multivariate analysis (MVA) adjusting for the time between end of radiotherapy and surgery in the subset of the population where this time was known. All tests were 2 sided, and *P* < .05 was considered significant. All computations were performed using SAS, version 9.4 (SAS Institute Inc).

## Results

A total of 34 500 patient records were reviewed; 21 679 patients (62.8%) were men and 12 821 (37.2%) were women, with a mean (SD) age at diagnosis of 59.7 (12.0) years. In terms of race and ethnicity, 2217 patients (6.4%) were Hispanic, 2843 (8.2%) were non-Hispanic Black, and 29 440 (85.3%) were non-Hispanic White (Figure 1). Differences were observed between non-Hispanic Black compared with non-Hispanic White patients by mean (SD) age at diagnosis (57.9 [11.5] and 61.1 [12.0] years, respectively; *P* < .001), sex (18 568 [63.1%] and 1678 [59.0%] men, respectively; *P* < .001), facility location (eg, large metropolitan area, 13 040 [44.3%] and 1726 [60.7%], respectively; *P* < .001), hospital volume (eg, ≥36, 18 984 [64.5%] and 1718 [60.4%] total LARC cases, respectively; *P* < .001), and primary payer at diagnosis (eg, Medicaid, 1833 [6.2%] and 417 [14.7%], respectively; *P* < .001) (Table 1). Non-Hispanic Black patients also had lower levels of education (2085 [73.3%] and 13 406 [45.5%), respectively; *P* < .001) and income (1987 [69.9%] and 13 463 [45.7%], respectively; *P* < .001). Regarding clinical variables, non-Hispanic Black patients had more comorbidities than non-Hispanic White patients (678 [23.8%] and 6272 [21.3%], respectively; *P* = .002) and were more often treated with APR in preference to LAR (846 [29.8%] and 8192 [27.8%], respectively; *P* = .03). Additional observed differences between non-Hispanic Black and non-Hispanic White patients included tumor grade (eg, grade 3 and grade 4, 332 [11.7%] and 3001 [10.2%], respectively; *P* = .006), clinical tumor stage (eg, cT3, 2400 [84.4%] and 25 349 [86.2%], respectively; *P* = .02), clinical nodal stage (eg, cN2, 329 [11.6%] and 3064 [10.4%], respectively; *P* = .02), and surgical margins (eg, positive, 197 [6.9%] and 1643 [5.6%], respectively; *P* = .01). On univariate analysis (UVA), tumor downstaging (1389 of 2958 [47.0%] and 16 806 of 32 472 [51.8%]; *P* < .001) and pN0 status (1936 of 2951 [65.6%] and 22 275 of 32 395 [68.8%]; *P* < .001) were less often achieved for non-Hispanic Black compared with non-Hispanic White patients. A pCR was obtained less often for non-Hispanic Black patients (348 of 2958 [11.8%]) than for Hispanic (347 of 2313 [15.0%]; *P* = .006) and non-Hispanic White (4795 of 32 472 [14.8%]; *P* < .001) patients (Table 2).

**Table 1.** Demographic, Clinical, and Pathologic Characteristics and Social Determinants of Health by Race and Ethnicity^a^

Characteristic	All (N = 34 500)	Hispanic (n = 2217)	Non-Hispanic Black (n = 2843)	Non-Hispanic White (n = 29 440)	*P* value		
					Non-Hispanic Black compared with non-Hispanic White	Hispanic compared with non-Hispanic White	Hispanic compared with non-Hispanic Black
Sex							
Male	21 679 (62.8)	1432 (64.6)	1678 (59.0)	18 569 (63.1)	<.001	.15	<.001
Female	12 821 (37.2)	785 (35.4)	1165 (41.0)	10 871 (36.9)			
Age at diagnosis, mean (SD), y	59.7 (12.0)	57.0 (11.9)	57.9 (11.5)	61.1 (12.0)	<.001	<.001	.007
Facility location							
Large metropolitan	16 177 (46.9)	1411 (63.6)	1726 (60.7)	13 040 (44.3)	<.001	<.001	<.001
Small metropolitan	10 861 (31.5)	615 (27.7)	744 (26.2)	9502 (32.3)			
Suburban	3970 (11.5)	102 (4.6)	218 (7.7)	3650 (12.4)			
Rural	2386 (6.9)	53 (2.4)	77 (2.7)	2256 (7.7)			
Unknown	1106 (3.2)	36 (1.6)	78 (2.7)	992 (3.4)			
Hospital volume, total LARC cases							
1-7	1228 (3.6)	101 (4.6)	137 (4.8)	990 (3.4)	<.001	<.001	.001
8-16	3567 (10.3)	275 (12.4)	260 (9.1)	3032 (10.3)			
17-35	7749 (22.5)	587 (26.5)	728 (25.6)	6434 (21.9)			
≥36	21 956 (63.6)	1254 (56.6)	1718 (60.4)	18 984 (64.5)			
Level of education							
High	17 369 (50.3)	577 (26.0)	758 (26.7)	16 034 (54.5)	<.001	<.001	.61
Low	17 131 (49.7)	1640 (74.0)	2085 (73.3)	13 406 (45.5)			
Level of income							
High	17 841 (51.7)	1008 (45.5)	856 (30.1)	15 977 (54.3)	<.001	<.001	<.001
Low	16 659 (48.3)	1209 (54.5)	1987 (69.9)	13 463 (45.7)			
Primary payer at diagnosis							
Private	17 689 (51.3)	902 (40.7)	1231 (43.3)	15 556 (52.8)	<.001	<.001	<.001
Medicaid	2684 (7.8)	434 (19.6)	417 (14.7)	1833 (6.2)			
Medicare	11 735 (34.0)	539 (24.3)	848 (29.8)	10 348 (35.1)			
Uninsured	1546 (4.5)	296 (13.4)	245 (8.6)	1005 (3.4)			
Other government	469 (1.4)	16 (0.7)	68 (2.4)	385 (1.3)			
Unknown	377 (1.1)	30 (1.4)	34 (1.2)	313 (1.1)			
Comorbidities							
Yes	7427 (21.5)	477 (21.5)	678 (23.8)	6272 (21.3)	.002	.81	.05
No	27 073 (78.5)	1740 (78.5)	2165 (76.2)	23 168 (78.7)			
Type of surgery							
Low anterior resection	24 893 (72.2)	1648 (74.3)	1997 (70.2)	21 248 (72.2)	.03	.03	.001
Abdominal perineal resection	9607 (27.8)	569 (25.7)	846 (29.8)	8192 (27.8)			
Dose of radiation, Gy							
<45.0	280 (0.8)	12 (0.5)	14 (0.5)	254 (0.9)	.004	.04	.93
45.0-50.4	8111 (23.5)	556 (25.1)	724 (25.5)	6831 (23.2)			
>50.4	26 109 (75.7)	1649 (74.4)	2105 (74.0)	22 355 (75.9)			
Neoadjuvant chemotherapy							
Yes	33 313 (96.6)	2136 (96.3)	2752 (96.8)	28 425 (96.6)	.49	.61	.38
No	1187 (3.4)	81 (3.7)	91 (3.2)	1015 (3.4)			
Surgical margins							
Positive	1977 (5.7)	137 (6.2)	197 (6.9)	1643 (5.6)	.01	.49	.54
Negative	32 125 (93.1)	2054 (92.6)	2610 (91.8)	27 461 (93.3)			
Unknown	398 (1.2)	26 (1.2)	36 (1.3)	336 (1.1)			
Tumor grade							
1	2553 (7.4)	184 (8.3)	205 (7.2)	2164 (7.4)	.006	.25	.23
2	23 498 (68.1)	1478 (66.7)	1867 (65.7)	20 153 (68.5)			
3 and 4	3567 (10.3)	234 (10.6)	332 (11.7)	3001 (10.2)			
Unknown	4882 (14.2)	321 (14.5)	439 (15.4)	4122 (14.0)			
Clinical tumor stage							
1	253 (0.7)	18 (0.8)	24 (0.8)	211 (0.7)	.02	.03	.83
2	1799 (5.2)	102 (4.6)	147 (5.2)	1550 (5.3)			
3	29 635 (85.9)	1886 (85.1)	2400 (84.4)	25 349 (86.1)			
4	2813 (8.2)	211 (9.5)	272 (9.6)	2330 (7.9)			
Clinical nodal stage							
0	14 805 (42.9)	844 (38.1)	1164 (40.9)	12 797 (43.5)	.02	<.001	.03
1	16 000 (46.4)	1071 (48.3)	1350 (47.5)	13 579 (46.1)			
2	3695 (10.7)	302 (13.6)	329 (11.6)	3064 (10.4)			

Abbreviation: LARC, locally advanced rectal cancer.

^a^ Unless otherwise indicated, data are expressed as No. (%) of patients. Percentages have been rounded and may not total 100.

**Table 2.** Univariate Analysis of Outcome by Race and Ethnicity^a^

Outcome	All	Hispanic	Non-Hispanic Black	Non-Hispanic White (n = 32 472)	*P* value		
					Non-Hispanic Black compared with non-Hispanic White	Hispanic compared with non-Hispanic White	Hispanic compared with non-Hispanic Black
pCR							
Yes	5490 (14.5)	347 (15.0)	348 (11.8)	4795 (14.8)	<.001	.66	.006
No	32 253 (85.5)	1966 (85.0)	2610 (88.2)	27 677 (85.2)			
Downstaging							
Yes	19 377 (51.3)	1131 (48.9)	1389 (47.0)	16 806 (51.8)	<.001	.01	.15
No	18 366 (48.7)	1182 (51.1)	1569 (53.0)	15 666 (48.2)			
Pathologic N0 status							
Yes	25 755 (68.4)	1544 (66.8)	1936 (65.6)	22 275 (68.8)	<.001	.02	.55
No	11 901 (31.6)	766 (33.2)	1015 (34.4)	10 120 (31.2)			

Abbreviation: pCR, pathologic complete response.

^a^ Unless otherwise indicated, data are expressed as No. (%) of patients.

Hispanic patients resembled non-Hispanic Black patients demographically. Differences were observed between Hispanic and non-Hispanic White patients by mean (SD) age at diagnosis (57.0 [11.9] and 61.1 [12.0] years, respectively; *P* < .001), facility location (eg, large metropolitan area, 1411 [63.6%] and 13 040 [44.3%], respectively; *P* < .001), hospital volume (eg, ≥36, 1254 [56.6%] and 18 984 [64.5%] total LARC cases, respectively; *P* < .001), and primary payer at diagnosis (eg, Medicaid, 434 [19.6%] and 1833 [6.2%], respectively; *P* < .001). Hispanic patients had lower levels of education (1640 [74.0%] and 13 406 [45.5%], respectively; *P* < .001) and income (1209 [54.5%] and 13 463 [45.7%], respectively; *P* < .001) compared with non-Hispanic White patients. As with non-Hispanic Black patients, Hispanic patients were treated with APR more often than non-Hispanic White patients (569 [25.7%] and 8192 [27.8%], respectively; *P* = .03), and there were noted differences by tumor stage (eg, cT3, 1886 [85.1%] and 25 349 [86.1%], respectively; *P* = .03) and nodal involvement (eg, cN2, 302 [13.6%] and 3064 [10.4%], respectively; *P* < .001). In contrast to non-Hispanic Black patients, UVA demonstrated no difference between rates of pCR for Hispanic and non-Hispanic White patients (347 of 2313 [15.0%] and 4795 of 32 472 [14.8%], respectively; *P* = .66) (Table 2); however, Hispanic patients resembled non-Hispanic Black patients in less often achieving tumor downstaging (1131 of 2313 [48.9%] and 16 806 of 32 472 [51.8%], respectively; *P* = .01) and pN0 status (1544 of 2310 [66.8%] and 22 275 of 32 395 [68.8%], respectively; *P* = .02) than non-Hispanic White patients.

**Figure 2.**
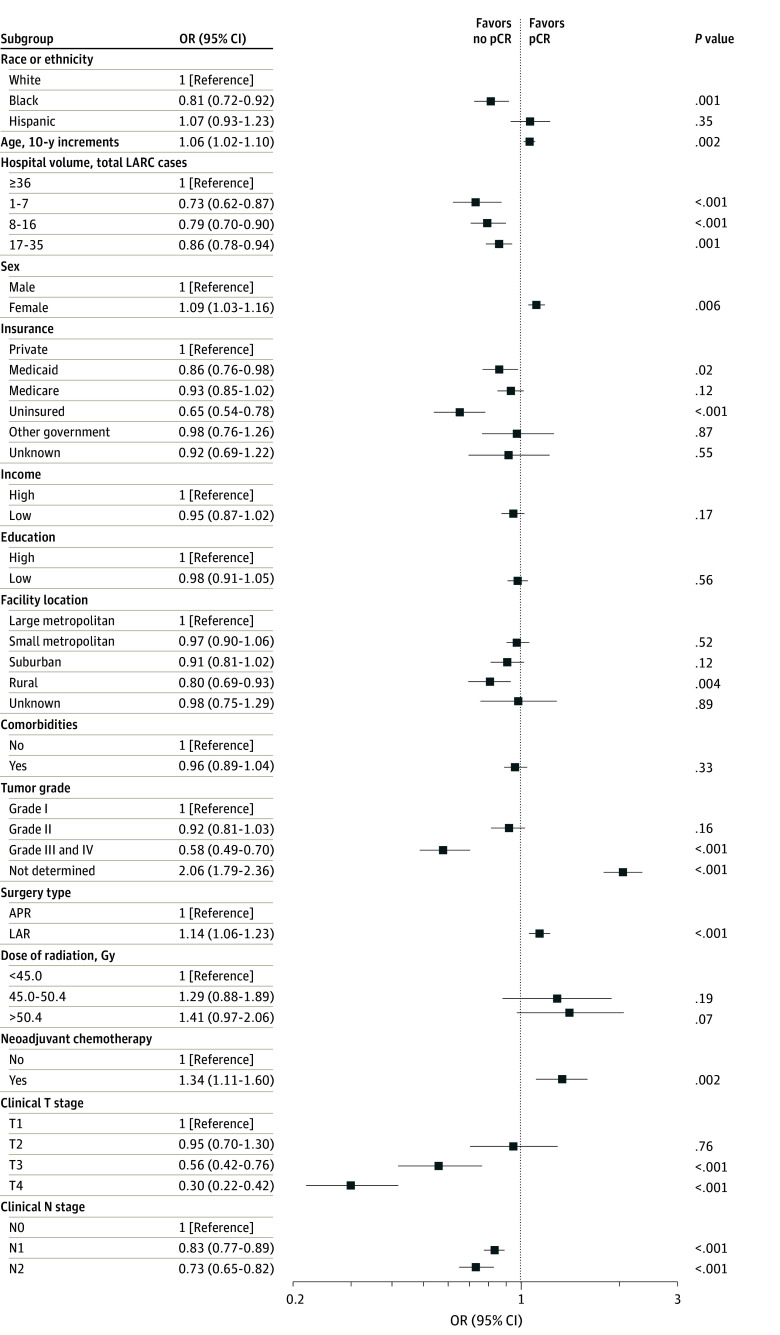
Multivariate Analysis of Pathologic Complete Response (pCR) Error bars indicate 95% CIs. APR indicates abdominoperineal resection; LAR, low anterior resection; LARC, locally advanced rectal cancer; and OR, odds ratio.

**Figure 3.**
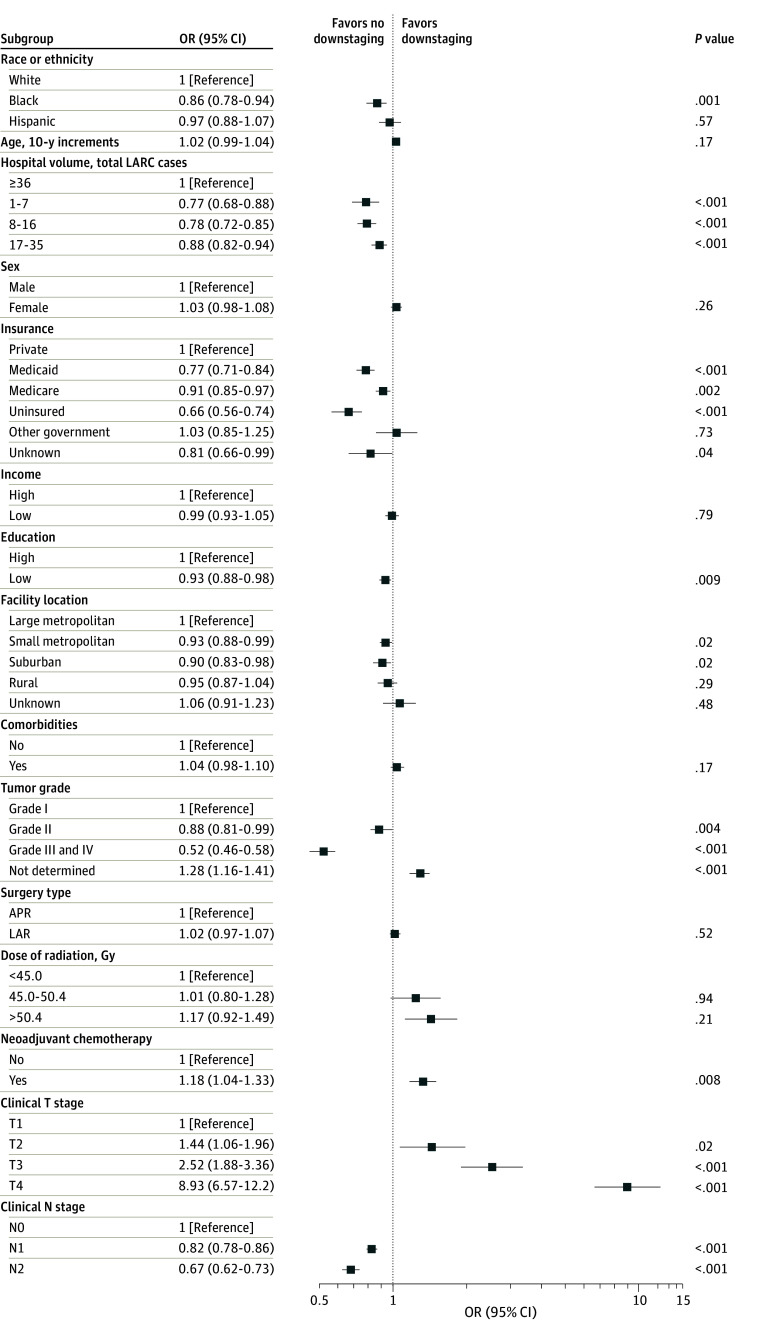
Multivariate Analysis of Tumor Downstaging Error bars indicate 95% CIs. APR indicates abdominoperineal resection; LAR, low anterior resection; LARC, locally advanced rectal cancer; and OR, odds ratio.

On MVA, a higher rate of pCR was associated with greater age (OR, 1.06 [95% CI, 1.02-1.10]) and female sex (OR, 1.09 [95% CI, 1.03-1.16]). Non-Hispanic Black race, but not Hispanic ethnicity, emerged as an independent risk factor for less frequent pN0 status (OR, 0.91 [95% CI, 0.83-0.99]) (eFigure in Supplement 1), less frequent pCR achievement (OR, 0.81 [95% CI, 0.72-0.92]) (Figure 2), and less frequent tumor downstaging (OR, 0.86 [95% CI, 0.78-0.94]) (Figure 3). Other risk factors for a reduced rate of pCR included a rural location (OR, 0.80 [95% CI, 0.69-0.93]), a lack of private medical insurance (OR for Medicaid, 0.86 [95% CI, 0.76-0.98]; OR for Medicare, 0.93 [95% CI, 0.85-1.02]; OR for no insurance, 0.65 [95% CI, 0.54-0.78]), and a treatment center with low patient volume (OR for first quartile, 0.73 [95% CI, 0.62-0.87]; OR for second quartile, 0.79 [95% CI, 0.70-0.90]; OR for third quartile, 0.86 [95% CI, 0.78-0.94]). Clinical and pathologic variables associated with a decreased pCR included higher tumor grade (OR, 0.58 [95% CI, 0.49-0.70]), more advanced tumor stage (OR for T3, 0.56 [95% CI, 0.42-0.76]; OR for T4, 0.30 [95% CI, 0.22-0.42]), and lymph node–positive disease (OR for N1, 0.83 [95% CI, 0.77-0.89]; OR for N2, 0.73 [95% CI, 0.65-0.82]). Neither area-level median income (OR, 0.95 [95% CI, 0.87-1.02]) nor area-level educational attainment (OR, 0.98 [95% CI, 0.91-1.05]) were associated with the likelihood of a pCR. In sensitivity analyses adjusting for time between completion of radiotherapy and surgery, associations between race or ethnicity and neoadjuvant treatment outcomes were similar to those of our main analysis (eTables 1-3 in Supplement 2).

## Discussion

This examination of a large multiethnic and multiracial cohort of patients with LARC undergoing neoadjuvant therapy and surgery confirmed and expanded on previously reported findings of racial and ethnic disparities in treatment outcome. We observed that Hispanic and non-Hispanic Black patients had primary higher-stage tumors with greater nodal involvement. Additionally, both Hispanic and non-Hispanic Black patients achieved less tumor downstaging and pN0 status than non-Hispanic White patients. Non-Hispanic Black patients, however, demonstrated a lower rate of pCR than Hispanic and non-Hispanic White patients. Both Hispanic and non-Hispanic Black patients with LARC were more likely to be treated with APR than non-Hispanic White patients and were thus less likely to undergo sphincter-preserving surgery. Both Hispanic and non-Hispanic Black patients achieved less tumor downstaging and pN0 status than non-Hispanic White patients. Only non-Hispanic Black patients, however, demonstrated a lower rate of pCR and continued to demonstrate an association with downstaging and pN0 status in adjusted MVA models. Of note, a sensitivity analysis that additionally adjusted for the time between completion of radiotherapy and surgery in the study population showed that inclusion of these variables did not change the interpretation of our findings.

To better understand the association of SDOH with outcomes, multiple other variables were examined. In this study, Hispanic and non-Hispanic Black patients were both distinguished from non-Hispanic White patients by their younger age, their lower educational and income levels, their frequent residence in metropolitan areas, their greater reliance on low-volume treatment centers, and their lack of private insurance. Prior work^53,54^ has consistently demonstrated the significant effect of insurance coverage on cancer outcomes. For instance, in an analysis conducted by Sineshaw et al^53^ involving Black and White patients with colorectal cancer in the NCDB, insurance coverage played a pivotal role, accounting for approximately one-half of the survival disparities observed between Black and White patients. The importance of insurance coverage extends throughout the entire cancer care journey, from diagnosis to the entirety of the treatment continuum.^53^ In our findings, we observed that in some cases, the distinguishing features of the Hispanic and non-Hispanic Black groups bore no obvious relation to the observed unfavorable clinical outcomes. A rural address, for example, has been associated with compromised health care access and reduced LARC survival.^20^ Urban areas, in contrast, are typically home to the high-volume centers that reportedly yield the highest rates of survival for LARC.^55^ In this study, Hispanic and non-Hispanic Black patients were found to live disproportionately in metropolitan areas but were more likely to be treated in low-volume centers, an anomaly that is perhaps due to transportation difficulties related to limited economic resources and/or lack of public transit to high-volume centers in residentially segregated areas.^27,29^ Low-volume centers often have limited resources that could influence clinical care. For instance, although APR and LAR procedures have been shown to be comparable with respect to local recurrence rates,^56^ LAR is more frequently performed in higher-volume settings where surgeons are more experienced or have been trained in colorectal surgery.^57,58^ Thus, it is possible that SDOH measures in this study could help explain differences in cN0 status and downstaging between Hispanic and non-Hispanic White patients, but they did not fully attenuate the association among non-Hispanic Black race, downstaging, and pCR.

To our knowledge, this is the first study in LARC to examine the association of multiple SDOH and demographic, clinical, and pathologic factors known to be predictive factors for treatment response. It is noteworthy that non-Hispanic Black race remained associated with an unfavorable treatment outcome, even after adjustment for the large number of SDOH and demographic, clinical, and pathologic variables included in this analysis. Particularly for non-Hispanic Black patients, evidently undiscovered factors contribute to their disparate outcomes. The lower frequency of pCR observed for non-Hispanic Black patients with LARC is consistent with other studies^59,60,61,62,63,64^ that have examined overall survival for non-Hispanic Black patients and reported (as is true for pCR) that patients who belong to ethnic and racial minority groups experience disproportionately unfavorable outcomes in terms of overall survival. Such reports provide a sound rationale for a follow-up to the current study that adopts overall survival as the primary outcome measure. Furthermore, the advanced stage and greater lymph node involvement observed in Hispanic and non-Hispanic Black patients raise questions about potential delays in diagnosis and their effect on the perceived surgical options at the time of diagnosis. Tumor location within the rectum may also play a role in these disparities. This suggests that improvements in cancer screening among these populations may be a viable strategy for diminishing the disparate treatment outcomes we observed for pN0 status and downstaging. Additionally, genetic differences associated with the non-Hispanic Black population are another potential undetected contributor to the observed disparities.^65^ As noted previously, investigation of genetic biomarkers has been impeded by underrepresentation of both Black and Hispanic patients in clinical studies and in major databases.^66^ Overall, while this study has underscored the persistence of disparities, particularly among non-Hispanic Black patients, it also emphasizes the urgent need for further investigations. Given our findings, any future study will need to follow the present one in considering the contribution of a wide range of demographic, socioeconomic, clinical, biological, and pathologic covariables. Ultimately, the goal is to enhance treatment outcomes for all patients, irrespective of their racial or ethnic backgrounds.

### Limitations

Study limitations arise from the unavailability of comprehensive data on all SDOH, including the social and community context domain, and biological factors (eg, genetics) that can contribute to clinical outcomes. Additional limitations include the retrospective nature of database research and the exclusion of cases with missing first-line treatment, race and ethnicity, or survival data. Exploring the effect of short- vs long-course radiotherapy is an interesting avenue for future research, and it could provide valuable information regarding its role in influencing treatment outcomes and demographic disparities, though it was outside the scope of this study. Given our large sample size, there is a possibility of a type I error, which can result in statistically significant findings that may lack clinical relevance; thus, the results should be carefully interpreted. The availability of genetic ancestry information in addition to self-reported race and ethnicity coding would allow a more comprehensive understanding of the complex problem investigated and the role of biology. Even after adjustment for available social, clinical, and demographic variables, non-Hispanic Black patients were more likely to have a suboptimal response to therapy. This could be attributable to other important, unmeasured SDOH, including measures of perceived discrimination and mistrust of the medical profession among non-Hispanic Black patients due to systemic racism in a US health care system with a history that includes past discriminatory treatment.^67,68,69,70,71,72^ Discrimination is challenging to capture in hospital-based datasets and is another study limitation. A national history of discriminatory practices, systemic inequalities, and structural barriers often due to policies (eg, redlining) have led to differences in SDOH, mistrust of the medical system by Black patients, and unconscious biases from physicians that could affect selection of treatment protocol, and ultimately treatment outcomes, particularly for non-Hispanic Black patients.^67,68,69,70,71,72^ We also recognize the intricate diversity and limitations inherent in the conventional racial and ethnic classifications used in this study. Self-reported race and ethnicity serve as oversimplified categorizations for multifaceted variations that encompass a range of intersecting factors such as ancestry and sociocultural influences.^73,74^ Additional possible explanations for the disparities in outcomes include either host or tumor factors that are associated with response to chemoradiotherapy. Not only are the clinical trials that define standard treatments composed overwhelmingly of White patients, but there are no widely available model systems to study this disease in Black patients. In future studies, it would be valuable to investigate potential disparities in LARC based on race and ethnicity by leveraging existing genomic data resources.

## Conclusions

In this cohort study, Hispanic and non-Hispanic Black patients receiving neoadjuvant therapy for LARC achieved lower rates of tumor downstaging and pN0 status than non-Hispanic White patients. For non-Hispanic Black patients only, pCR rates were also less than for non-Hispanic White patients. In a comparison with non-Hispanic White patients, Hispanic and non-Hispanic Black patients were younger, with lower levels of education and income and less frequent coverage by private health insurance, and had tumors of higher stage with greater nodal involvement. Despite living in metropolitan areas, they tended to receive treatment in lower-volume centers where they disproportionately underwent APR rather than sphincter-preserving surgery. After controlling for all covariables affecting treatment outcome, non-Hispanic Black race remained independently associated with a reduced likelihood of a pCR. The results suggest that the racial and ethnic disparities in treatment outcomes for LARC are complexly determined, encompassing multiple socioeconomic, clinical, and pathologic variables as well as additional unknown variables—including possible biological and SDOH differences—that must be unraveled before racial and ethnic disparities can be overcome.

## Notes

Supplement 1. **eFigure.** MVA of Pathologic N0 Status

Supplement 2. **eTable 1.** Sensitivity Analysis of pCR **eTable 2.** Sensitivity Analysis of Downstaging **eTable 3.** Sensitivity Analysis of pN0 Status

Supplement 3.
Data Sharing Statement
