# Supplementary material for: Factors Associated With Racial and Ethnic Disparities in Locally Advanced Rectal Cancer Outcomes
Source: JAMA Netw Open. 2024 Feb 29;7(2):e240044. doi: 10.1001/jamanetworkopen.2024.0044 (PMC10905315; doi:10.1001/jamanetworkopen.2024.0044)
Supplement: Supplement 1. — eFigure. MVA of Pathologic N0 Status [file jamanetwopen-e240044-s001.pdf]

Supplementary Online Content

Shulman RM, Deng M, Handorf EA, Meyer JE, Lynch SM, Arora S. Factors contributing to racial and ethnic disparities in locally advanced rectal cancer outcomes. *JAMA Netw Open*. 2024;7(2):e240044. doi:10.1001/jamanetworkopen.2024.0044

eFigure. MVA of Pathologic N0 Status
eTable 1. Sensitivity Analysis of pCR
eTable 2. Sensitivity Analysis of Downstaging
eTable 3. Sensitivity Analysis of pN0 Status

This supplementary material has been provided by the authors to give readers additional information about their work.

© 2024 Shulman RM et al. *JAMA Network Open*.

eFigure. MVA of Pathologic N0 Status

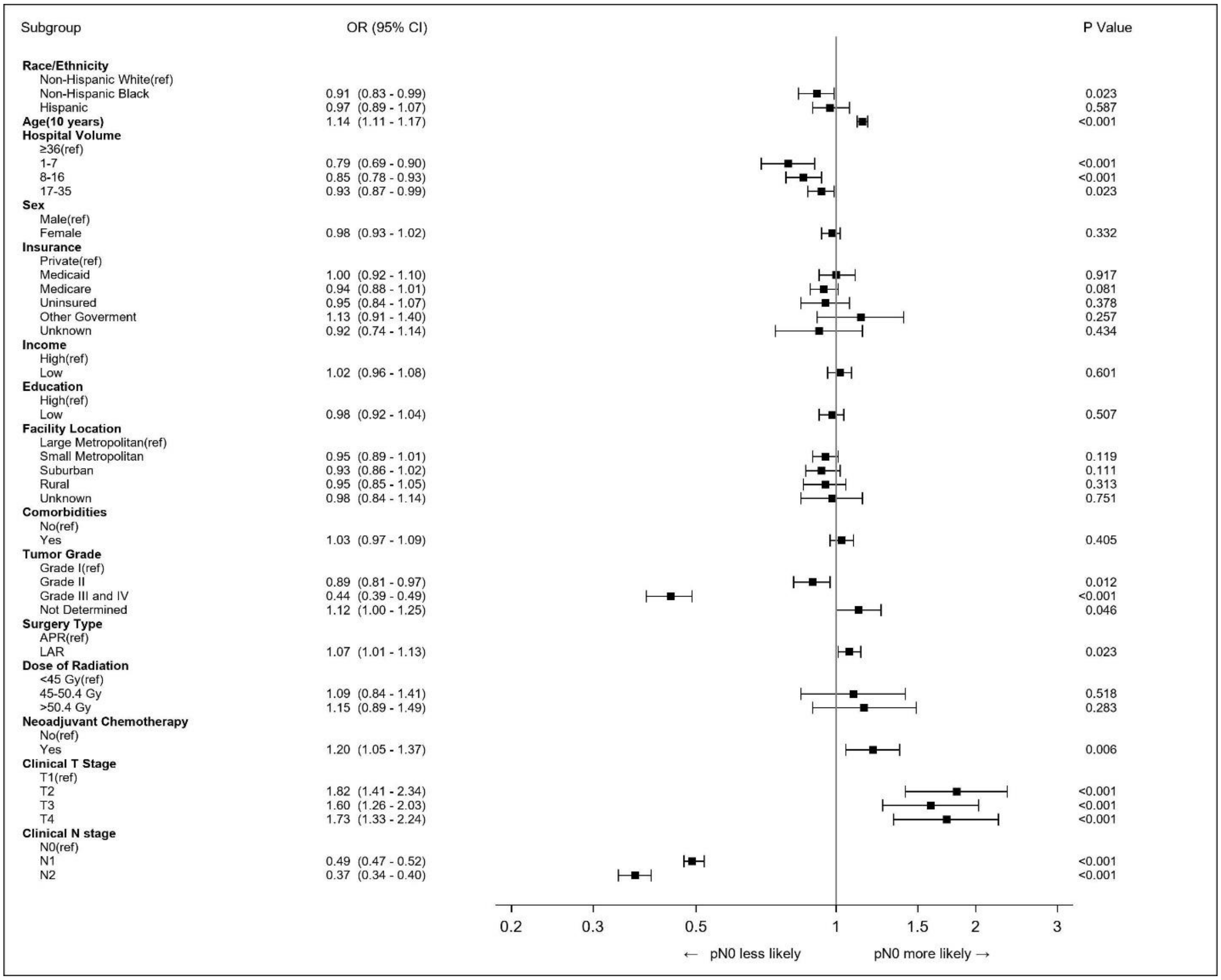

Note: pN0 associations with clinical T-stage are driven partially by the study inclusion criteria (cT1-2/N0 are not locally advanced and are excluded from the cohort).

© 2024 Shulman RM et al. *JAMA Network Open*.
