# Supplementary material for: Factors Associated With Racial and Ethnic Disparities in Locally Advanced Rectal Cancer Outcomes
Source: JAMA Netw Open. 2024 Feb 29;7(2):e240044. doi: 10.1001/jamanetworkopen.2024.0044 (PMC10905315; doi:10.1001/jamanetworkopen.2024.0044)
Supplement: Supplement 3. — Data Sharing Statement [file jamanetwopen-e240044-s003.pdf]

Data Sharing Statement

Shulman. Factors Contributing to Racial and Ethnic Disparities in Locally Advanced Rectal Cancer Outcomes. *JAMA Netw Open*. Published February 29, 2024.
doi:10.1001/jamanetworkopen.2024.0044

Data
Data available: No

Additional Information
Explanation for why data not available: The data used in the study are derived from a de-identified National Cancer Database (NCDB) file. The American College of Surgeons and the Commission on Cancer have not verified and are not responsible for the analytic or statistical methodology employed, or the conclusions drawn from these data by the investigator. The study analyzed data from the NCDB, which collects de-identified data of cancer patients across the United States. Authors are prohibited to share the data per data use agreement, but interested parties could apply for the data directly from the NCDB.
